# Metabolic fingerprinting of the Antarctic cyanolichen *Leptogium puberulum*–associated bacterial community (Western Shore of Admiralty Bay, King George Island, Maritime Antarctica)

**DOI:** 10.1007/s00248-021-01701-2

**Published:** 2021-02-08

**Authors:** Jakub Grzesiak, Aleksandra Woltyńska, Marek K. Zdanowski, Dorota Górniak, Aleksander Świątecki, Maria A. Olech, Tamara Aleksandrzak-Piekarczyk

**Affiliations:** 1 Institute of Biochemistry and Biophysics, Polish Academy of Sciences, Pawińskiego 5A, 02-106 Warszawa, Poland; 2 Department of Microbiology and Mycology, University of Warmia and Mazury in Olsztyn, Oczapowskiego 1a, 10-719 Olsztyn, Poland; 3 Institute of Botany, Jagiellonian University, Gronostajowa 3, 30-387 Krakow, Poland

**Keywords:** EcoPlates, Microbiome, Symbiosis, Metabolism, Bacteria, Lichens

## Abstract

Lichens are presently regarded as stable biotopes, small ecosystems providing a safe haven for the development of a diverse and numerous microbiome. In this study, we conducted a functional diversity assessment of the microbial community residing on the surface and within the thalli of *Leptogium puberulum*, a eurytopic cyanolichen endemic to Antarctica, employing the widely used Biolog EcoPlates which test the catabolism of 31 carbon compounds in a colorimetric respiration assay. Lichen thalli occupying moraine ridges of differing age within a proglacial chronosequence, as well as those growing in sites of contrasting nutrient concentrations, were procured from the diverse landscape of the western shore of Admiralty Bay in Maritime Antarctica. The *L. puberulum* bacterial community catabolized photobiont- (glucose-containing carbohydrates) and mycobiont-specific carbon compounds (d-Mannitol). The bacteria also had the ability to process degradation products of lichen thalli components (d-cellobiose and *N*-acetyl-d-glucosamine). Lichen thalli growth site characteristics had an impact on metabolic diversity and respiration intensity of the bacterial communities. While high nutrient contents in lichen specimens from “young” proglacial locations and in those from nitrogen enriched sites stimulated bacterial catabolic activity, in old proglacial locations and in nutrient-lacking sites, a metabolic activity restriction was apparent, presumably due to lichen-specific microbial control mechanisms.

## Introduction

The term Linnaeus used in 1775 to describe lichens was “poor trash of vegetation;” however, this could not be further from the truth [1]. Lichens are exemplary in showcasing symbiosis between their two main components: a mycobiont and a photobiont [2–4]. These organisms associate into easily recognizable and species-specific structures—the vegetative thalli [2]. They thrive in almost all terrestrial habitats on Earth, covering up to 8% of land surface [1], and are among the first to colonize extreme habitats and newly exposed land [5]. Lichens are more successful in cold habitats than vascular plants; thus, the terrestrial vegetation of ice-free regions of Maritime and Continental Antarctica is dominated by them [6].

Studies on Antarctic lichens revealed their vast distribution, as well as their strict requirements for particular environmental conditions [7]. It has been concluded that nitrogen is a major factor governing the growth of lichens, with species occurrence being strongly linked to nutrient gradients, caused mainly by old and contemporary penguin nesting sites [6, 8]. According to nitrogen compound concentration preference, lichen species can be: nitrophilous (thriving in nutrient rich sites, irrespective of other environmental variables), nitrogen-sensitive (avoiding high nitrogen concentrations) or nitrogen-tolerant (growing regardless of nitrogen compound concentrations) [9, 10]. Furthermore, lichens actively participate in primary succession following deglaciation events, ever so accelerating due to global warming [11]. Along with bryophytes, lichens are considered key organisms in the development of the Antarctic terrestrial ecosystem [10].

Non-photobiont prokaryotes, frequently observed on the surface and within the lichen thalli, have been dismissed as functionally irrelevant or even environmental contaminants. However, the dawn of molecular microbiology techniques has led to a recognition of lichen thalli as stable biotopes, small ecosystems providing a safe haven for the development of a diverse and numerous bacteriocenosis [12]. Lichen-hosted bacterial communities have been investigated in numerous lichen species, yet there are no comprehensive studies on if, and how they change, depending on nutrient content preference of the host lichen, as well as the thalli situation within a proglacial chronosequence.

To elucidate if such changes really do occur, we investigated the microbiome associated with the Antarctic lichen *Leptogium puberulum* Hue, a bipartite, foliose lichen, with *Nostoc* cyanobacteria serving as its photobiont. Like all lichen cyanobionts, *Nostoc* cells are located in the lichen thalli extracellularly and possess nitrogen-fixing capabilities [2], making the lichen largely independent of external labile nitrogen sources. This lichen species resides both in nutrient-rich habitats surrounding penguin rookeries, as well as in nutrient-lacking areas [10, 13]. Maritime Antarctica, especially the western shore of Admiralty Bay, presents an excellent site for this type of research. On this relatively small area, sites experiencing high inputs of organic matter (marine bird nesting sites) border those with very limited nutrient content (glacier forefields and dry valleys) [14]. Therefore, these naturally forming trophic and spatio-temporal gradients (glacier foreland chronosequences) have been explored in this paper as study sites.

The main aim of this investigation was to assess carbon compound utilization patterns of the *L. puberulum* associated bacterial community in relation to lichen thalli habitat “age,” along with the exposure of its growth habitat to varying nutrient amounts. Our working hypothesis is that thalli situation within the diverse landscape of the study site has a profound impact on the lichen-hosted microbiome, which is reflected in its carbon source utilization abilities, in terms of diversity and intensity. We thus conducted a functional diversity assessment of the bacterial community residing on the surface and within the thalli of *L. puberulum*, employing the widely used Biolog EcoPlates, to shed some light on the mechanisms shaping the lichen-hosted bacterial communities in relation to its ecological niche. This is the first paper to tackle the topic of microbial metabolic activity in Antarctic lichens.

## Materials and Methods

The samples were obtained during the 43^rd^ Expedition to the Polish Antarctic Station “Arctowski” in late February/early March of 2019 from ice-free areas along the western shore of Admiralty Bay (King George Island, Antarctica), as well as the barren terrains that border the Southern shore of the Ezcurra Inlet [10]. Lichen specimen samples were collected into sterile containers with sterile tweezers and scissors in triplicate from each sampling site.

### Lichen Sampling Scheme

Samples of the Antarctic eurytopic cyanolichen *L. puberulum* were collected at four points within a transect on the foreland of the receding Ecology Glacier (King George Island, Maritime Antarctica). This “spatio-temporal gradient” reflected the recession of Ecology Glacier [15–17]. A transect was established that ran across lateral moraines. The first sampling point (L1) was the “youngest” site (time since exposure from beneath glacial ice), where *L. puberulum* growth was apparent. The time since exposure from beneath the ice was circa 20–30 years between sampling points (Table 1). The last sampling point (L4) was established on a ridge of a Neoglacial moraine that has been ice-free for at least 100 years. *L. puberulum* samples were also collected from two locations varying in nutrient availability. The nutrient-lacking area of Jardine Peak (Southern shore of the Ezcurra Inlet) and the nutrient-abundant area near the Point Thomas penguin rookery were chosen as sampling sites to accommodate the “trophic gradient.” For comparative reasons, samples of the ornithocoprophilous/nitrophilous green algae-containing Antarctic lichen *Gondwania regalis* (Vain.) Søchting, Frödén, and Arup (former *Caloplaca regalis* (Vain.) Zahlbr.) were collected from the nutrient-abundant area near the Point Thomas penguin rookery. The samples were transported within one hour to the Polish Antarctic Station “Arctowski” and processed at the field laboratory. Taxonomic identification of lichen specimens was done by Maria A. Olech.

**Table 1:** Sampling site description

Sampling site	Site description	Approximate sampling site age (time since deglaciation)	Coordinates
***Spatio-temporal gradient***			
L1	Ecology Glacier foreland; contemporary lateral moraine ridge; mostlystones and gravel; very sparse vegetation; isolated colonies of *Leptogiumpuberulum* and *Sanionia uncinata* (moss), *Colobanthus quitensis* seedlings; single specimens of *Usnea antarctica* on stones	39 years	62°10′00.6 58°28'07.3
L2	Ecology Glacier foreland; contemporary lateral moraine ridge; mostlyloose stones and gravel, sparse vegetation, large colonies of *Leptogiumpuberulum*, isolated small colonies of *Colobanthus quitensis* and *Deschampsia antarctica*, larger isolated specimens of *Usnea antarctica*	46 years	62°09′58.7 58°28'05.7
L3	Ecology Glacier foreland; neoglacial lateral moraine ridge; gravel semi-bound by vegetation, loose community made up of *Leptogium puberulum*, *Usnea antarctica*, *Deschampsia antarctica* and several species of mosses	65 years	62°09′57.4 58°27′59.1
L4	Ecology Glacier foreland; neoglacial lateral moraine ridge; ground mostly covered by vegetation: *Leptogium puberulum*, *Deschampsia antarctica*, *Usnea antarctica* and other chlorolichen and moss species	>100 years	62°09′56.4 58°27′58.8
***Trophic gradient***			
LP	Point Thomas Penguin Rookery; flat planes covered with decaying penguin excreta and weathered basaltic rocks of varying height occupied by nitrophilous lichens(*Leptogium puberelum*, *Xanthoria* spp., *Caloplaca* spp. and others	N/A	62°09′47.8 58°27′32.5
CR		N/A	62°09′48.1 58°27′36.8
LJ	Jardine Peak area; mostly loose rocks and gravel; large colonies of *Leptogium puberulum,* nitrophobic lichen communities on neighboring rock walls (*Usnea aurantiaco-atra*, *Himantormia lugubris*)	N/A	62°09′57.4 58°28′13.3

Spatio-temporal gradient – sampling site at the Ecology Glacier forefield across lateral moraines where the distance from the glaciers edge can be substituted for the time since deglaciation. Trophic gradient – sampling sites of varying nutrient concentrations (Point Thomas Penguin Rookery – high nutrient conc.; Jardine Peak area – low nutrient conc.)

### External Microbial Fraction Isolation from the Lichen Thallus

The lichen thalli were briefly rinsed with sterile water to rid them of soil and dust. 0.2 g of the rinsed thallus was placed in a 50 mL centrifuge tube containing 20 mL of extraction fluid (per 100 mL: 2.4 g mannitol, 3 g sorbitol, 0.05 g cysteine, 0.05 g ascorbic acid, 1 μl Tween80, 0.17 g Na_4_P_2_O_7̽_ 10 H_2_O) and incubated for 30 min at 4°C. The samples were then shaken for 1 h in a Tornado^TM^ Vortexer at 1000 rpm at 4 °C, then placed in a VWR Ultrasonic Cleaner USC-TH filled with chilled water and sonicated for 5 min and shortly vortexed afterwards. The extract was filtered into a new 50 mL centrifuge tube using a sterile cell strainer with a 70 μm mesh. The resulting suspension was used to inoculate the Biolog EcoPlates (Biolog Inc., Hayward, CA).

### Internal Microbial Fraction Isolation from the Lichen Thallus

The washed lichen thallus was cut into little pieces on a sterile Petri dish using a sterile scalpel and transferred into a sterile mortar, to which 3 mL of extraction fluid was added, as well as 0.5 g of sterile, sharp, garnet sand (Lysing Matrix A, MP Biomedicals). The samples were delicately ground using a sterile pestle until they had a uniform consistency and were transferred into a 50 mL centrifuge tube containing 17 mL of extraction fluid. The samples were then shaken for 30 min in a Tornado^TM^ Vortexer at 1000 rpm at 4 °C, then placed in a VWR Ultrasonic Cleaner USC-TH filled with chilled water and sonicated for 5 min and shortly vortexed afterwards. To separate the extract, the material was centrifuged (60 s, 1000 RPM, 4 °C). Ten milliliters of the upper supernatant fraction was transferred into a 15 mL centrifuge tube. The resulting suspension was used to inoculate the Biolog EcoPlates.

### Phenotype Fingerprinting with Biolog EcoPlate™

Lichen-associated bacterial suspensions were centrifuged at 6000 rpm for 5 min at 4 °C, suspended in sterile, cool 0.9% saline supplemented with nystatin (final concentration 50 μg/mL) to prevent fungal metabolism, and adjusted with sterile 0.9% saline to the optical transmittance of 0.9. One hundred microliters aliquots of each suspension were added to each well of EcoPlate microtiter plates. EcoPlates contain 3 repeated sets of 31 carbon sources and employ a tetrazolium redox dye as an indicator of microbial metabolism. As microbes utilize the carbon sources, they respire and the tetrazolium reporter dye is reduced to form a visible purple color. Communities of microorganisms will exhibit a characteristic reaction pattern, a metabolic fingerprint that reflects the metabolic properties of the community. One plate (which contains 3 replicates) has been used per suspension (42 plates were used in total). The plates were incubated in darkness at 4 °C, with the color development measured in an OmniLog microplate reader (Biolog Inc., Hayward, CA). Cellular respiration was measured kinetically by determining the colorimetric reduction of the tetrazolium dye. Data were collected approximately twice a week over a 65 day period. The Biolog EcoPlate assays assess the ability of a mixed microbial community to utilize any of the 31 carbon compounds as the sole carbon source (plus a single control well without a carbon source). Microbial communities were characterized by their ability to catabolize 10 different carbohydrates, 9 carboxylic and acetic acids, 4 polymers, 6 amino acids, and 2 amines [18]. Data from the 42^nd^ day of incubation was used, as there was no further color development after this date. The final absorbance was first blanked against the “zero” reading time and then blanked against the respective control well without a carbon source. Obtained colorimetric measurement values are given as Omnilog Arbitrary Units (OAU).

### Data Analysis

All results were compiled using Excel (MS Office) 2016 for Windows. Data visualization and statistical analysis have been performed using the R software (R version 4.0.2) and the following packages: ggplot2, fmsb, Hmisc, corrplot, and autoplot.

## Results

Carbon source utilization of the cyanolichen *L. puberulum* associated bacterial community was assessed on the Biolog EcoPlate tetrazolium salt reduction assay. Sample identities were as follows: L, *L. puberulum*; CR, *G. regalis*; E, external community; I, internal community; L1-L4, *L. puberulum* samples from the Ecology Glacier foreland; P, *L. puberulum* samples from the Point Thomas penguin rookery; and J, *L. puberulum* samples from the Jardine Peak area.

**Fig. 1:**
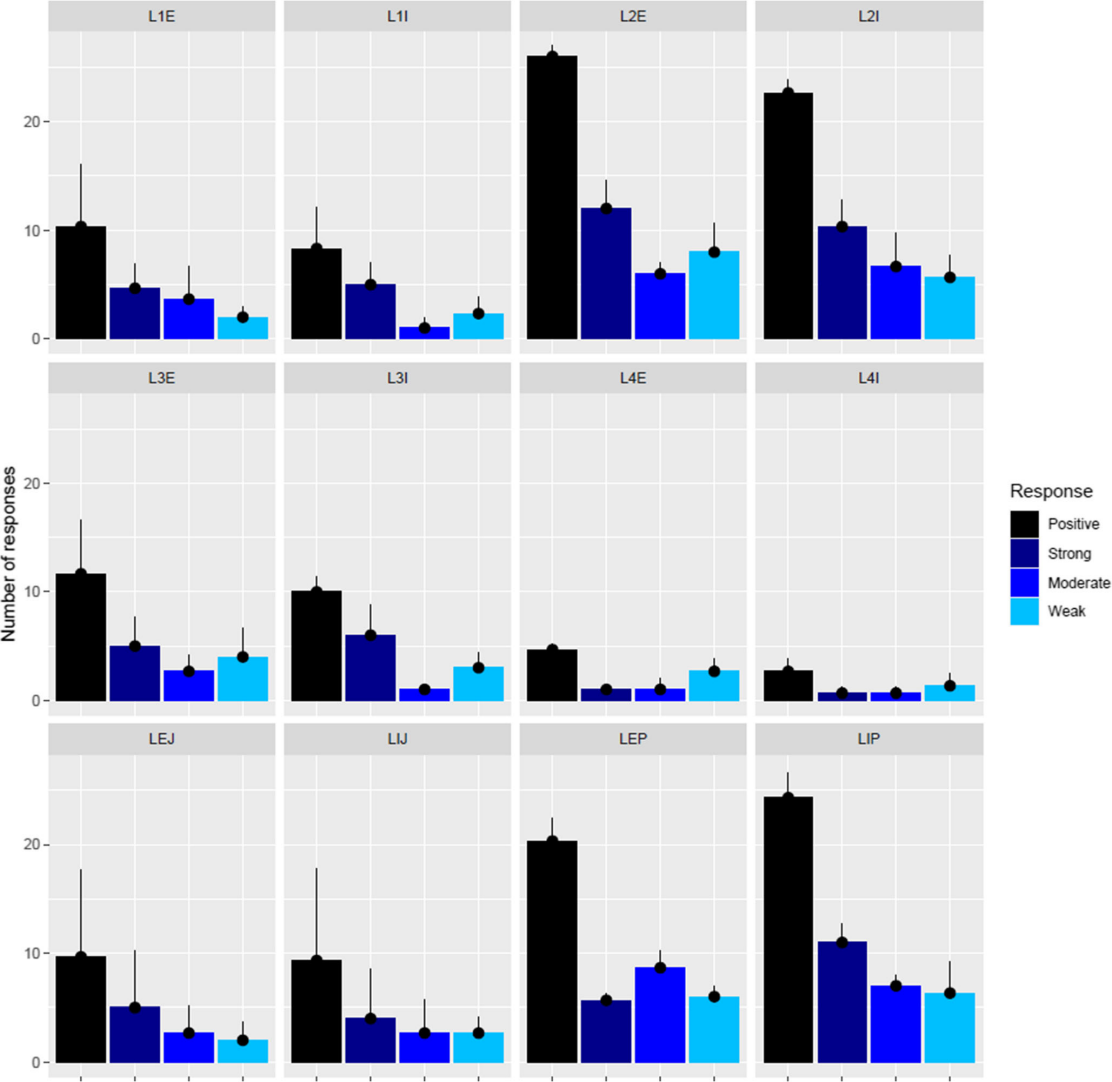
Response numbers of *Leptogium puberulum* associated bacterial community based on the Biolog EcoPlate carbon source utilization colorimetric assay coupled with tetrazolium salt reduction. E, external community; I, internal community; L1-L4, *Leptogium puberulum* samples of the Ecology Glacier foreland; P, *Leptogium puberulum* samples of Point Thomas penguin rookery; J, *Leptogium puberulum* samples of Jardine Peak area. Positive, colorimetric response >50 Omnilog Arbitrary Units; strong, colorimetric response >150 Omnilog Arbitrary Units; moderate, colorimetric response = 100–150 Omnilog Arbitrary Units; weak, colorimetric response = 50–100 Omnilog Arbitrary Units

OAU values were in the range of 0.00–233.67. The number of responses at a cut-off value of ≥ 50 OAU (regarded as positive responses) was the highest in samples: L2E (av. 26.0±1.00) responses), LIP (av. 24.33±2.31 responses), LEP (av. 20.33±2.08 responses), and L2I (av. 22.67±1.15 responses). The lowest positive response numbers were obtained in samples: L4E and L4I (av. 4.67±0.58 and av. 2.67±1.15 responses respectively). Similar values of positive response numbers were achieved in samples: L1E (av. 10.33±5.69), L1I (av. 8.33±3.79), L3E (av. 11.67±4.93), L3I (av. 10.00±1.41), LEJ (av. 9.67±8.08), and LIJ (av. 9.33±8.50). Strong responses (≥150 OAU) were seen to dominate in samples L2E (av. 12.00±2.65 responses) and LIP (av. 11±1.73 responses) (Fig. 1).

**Fig. 2:**
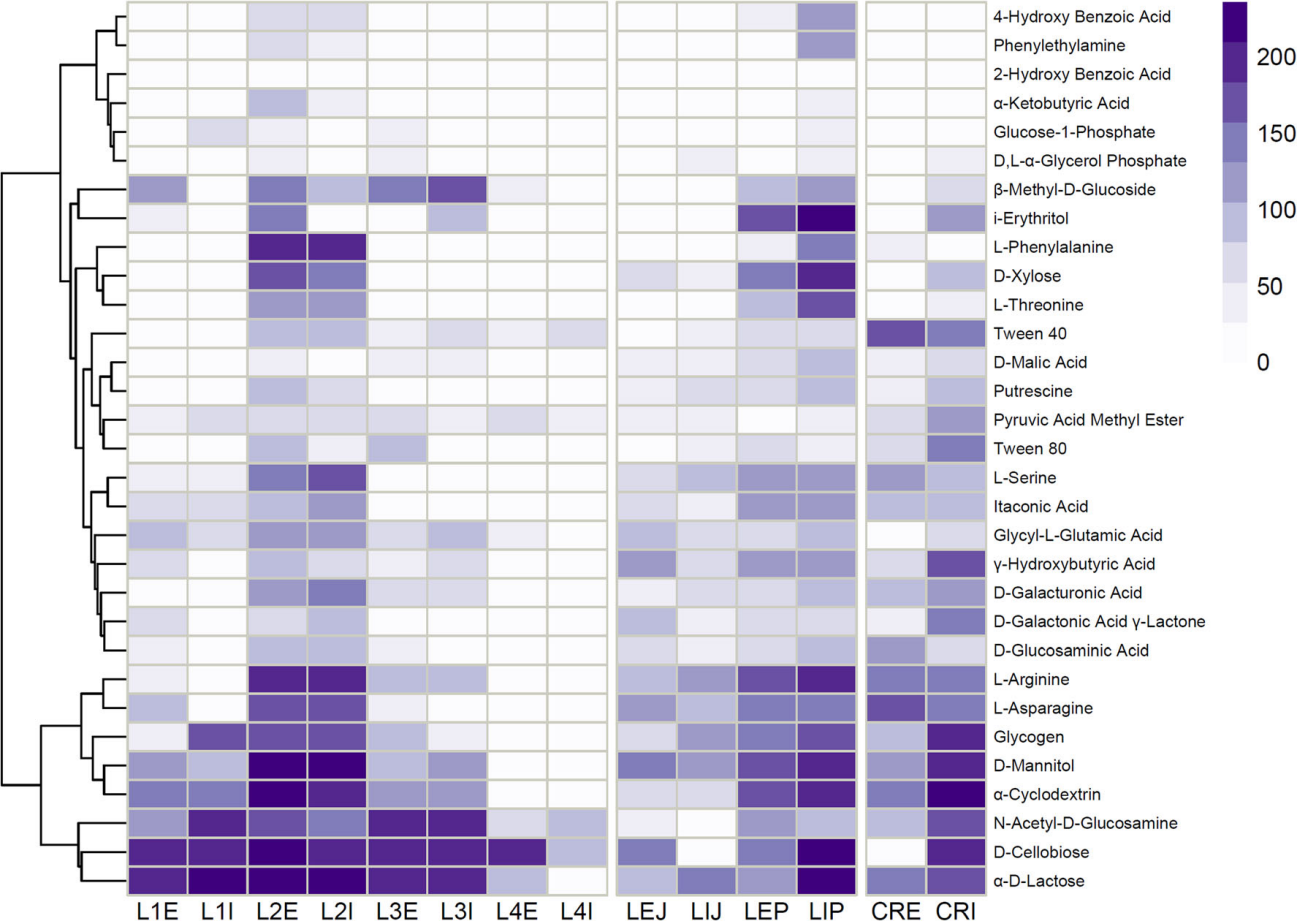
Heatmap displaying *Leptogium puberulum* bacterial community responses on Biolog EcoPlates. Color saturation represents carbon source metabolism intensity. Scale given in Omnilog Arbitrary Units. L, bacterial community of *Leptogium puberulum*; CR, bacterial community of *Caloplaca regalis*; E, external community; I, internal community; L1-L4, *Leptogium puberulum* samples of the Ecology Glacier foreland; P, *Leptogium puberulum* samples of Point Thomas penguin rookery; J, *Leptogium puberulum* samples of Jardine Peak area

Carbon sources utilized by the *L. puberulum* and *G. regalis* (added for comparison) associated bacterial communities belonged mostly to the carbohydrate related group: α-d-lactose (av. 164.6 OAU), d-cellobiose (av. 158.6 OAU), *N*-acetyl-d-glucosamine (av. 123.2 OAU), α-cyclodextrin (av. 129.5 OAU), d-Mannitol (av. 126.4 OAU), and glycogen (av. 103.4 OAU), as well as amino acids: l-asparagine (av. 92.6 OAU), l-arginine (av. 104.2 OAU) (Fig. 2).

**Fig. 3:**
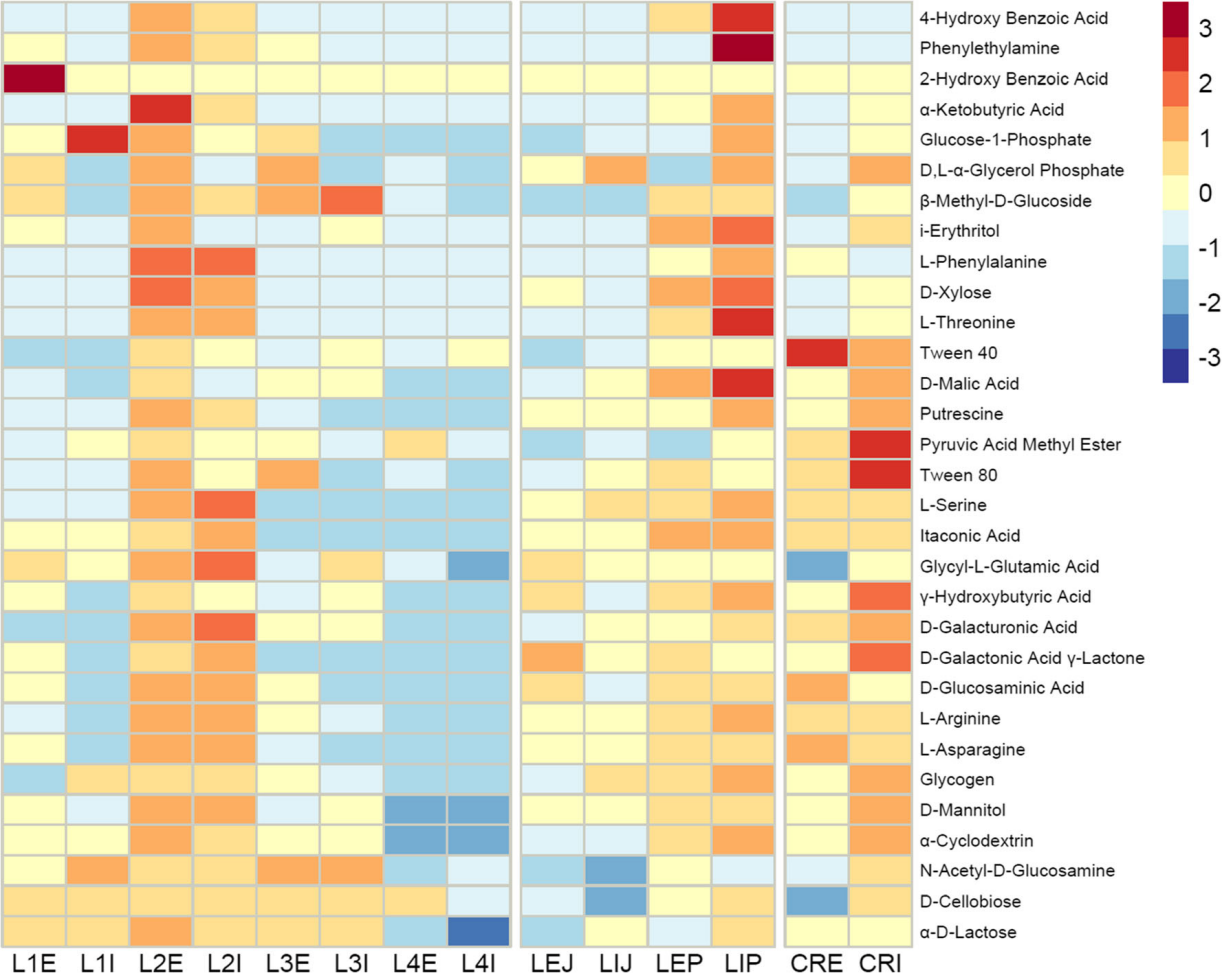
Heatmap displaying *Leptogium puberulum* bacterial community responses on Biolog EcoPlates. Scaling done within rows, indicating the highest and the lowest values across all samples within a particular carbon source. L, bacterial community of *Leptogium puberulum*; CR, bacterial community of *Caloplaca regalis*; E, external community; I, internal community; L1-L4, *Leptogium puberulum* samples of the Ecology Glacier foreland; P, *Leptogium puberulum* samples of Point Thomas penguin rookery; J, *Leptogium puberulum* samples of Jardine Peak area

The differences between the communities were apparent, as shown by the discrepancies in the utilization efficiency of several carbon sources (Fig. 3). Considerably high values within the same carbon source were obtained for the following samples: L1I (glucose-1-phosphate—64.8 OAU), L2E (α-ketobutyric acid—82.4 OAU, d-xylose—174 OAU, l-phenylalanine—195.5 OAU), L2I (l-phenylalanine—193.3 OAU, l-serine—167.4 OAU, glycyl-l-glutamic acid—120.9 OAU, d-galacturonic acid—151.6 OAU), L3I (β-methyl-d-glucoside—176.6 OAU), LIP (4-hydroxy benzoic acid -122.2 OAU, phenylethylamine—121.6 OAU, d-xylose—185.9 OAU, l-threonine—168.22 OAU, d-malic acid–101.2 OAU), CRE (Tween 40—175 OAU), and CRI (pyruvic acid methyl ester—128.6 OAU, Tween 80—147.7 OAU, γ-hydroxybutyric acid—160.6 OAU, d-galactonic acid γ-Lactone – 139.2 OAU). Unusually low efficiency, or even no utilization at all within the same carbon source, was noted in the following samples: L4E (d-Mannitol—0.0 OAU, α-cyclodextrin—5.4 OAU), L4I (d-Mannitol—0.0 OAU, α-cyclodextrin—0.0 OAU, α-d-lactose—9.7 OAU), LIJ (*N*-acetyl-d-glucosamine—16.7 OAU, d-cellobiose—1.0 OAU), and CRE (glycyl-l-glutamic acid—16.8 OAU, d-cellobiose—16.8 OAU).

**Fig. 4:**
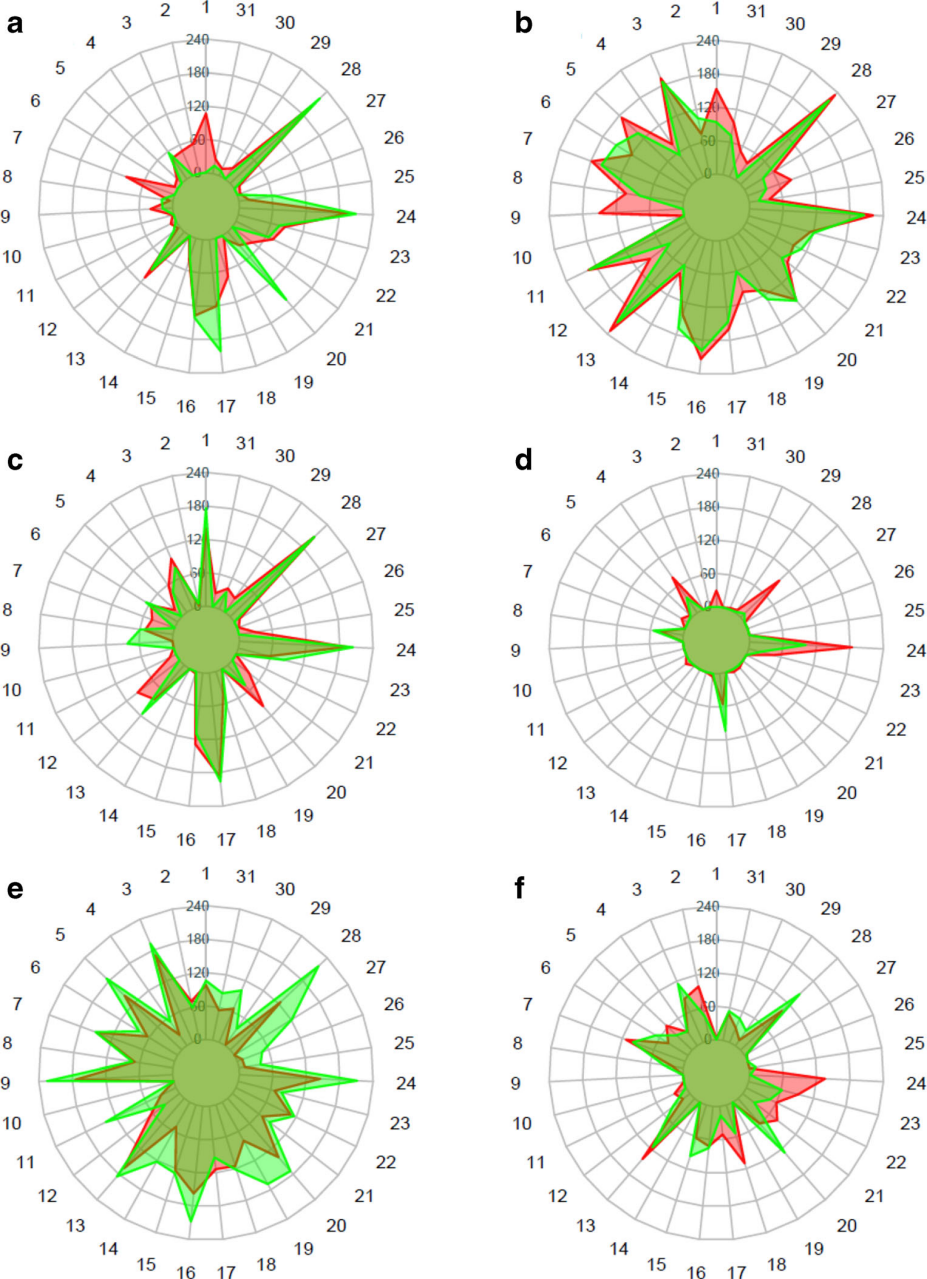
Radar charts of *Leptogium puberulum* bacterial community responses on Biolog EcoPlates. External community responses, red; internal community responses, green. **a** *Leptogium puberulum* bacterial community responses in sample L1 (Ecology Glacier foreland, closest to glacier terminus); **b** *Leptogium puberulum* bacterial community responses in sample L2 (Ecology Glacier foreland), **c** *Leptogium puberulum* bacterial community responses in sample L3 (Ecology Glacier foreland), **d** *Leptogium puberulum* bacterial community responses in sample L4 (Ecology Glacier foreland, farthest from the glacier terminus), **e** *Leptogium puberulum* bacterial community responses in sample LP (Point Thomas penguin rookery), **f** *Leptogium puberulum* bacterial community responses in sample LJ (Jardine Peak area). Scale given in Omnilog Arbitrary Units. 1, β-methyl-d-glucoside; 2, d -galactonic acid γ-lactone; 3, l-arginine; 4, pyruvic acid methyl ester; 5, d-xylose; 6, d-galacturonic acid; 7, l-asparagine; 8, Tween 40; 9, i-erythritol; 10, 2-hydroxy benzoic acid; 11, l-phenylalanine; 12, Tween 80; 13, d-Mannitol; 14, 4-hydroxy benzoic acid; 15, l-serine; 16, α-cyclodextrin; 17, *N*-acetyl-d-glucosamine; 18, γ-hydroxybutyric acid; 19, l-threonine; 20, glycogen; 21, d-glucosaminic acid; 22, itaconic acid; 23, glycyl-l-glutamic acid; 24, d-cellobiose; 25, glucose-1-phosphate; 26, α-ketobutyric acid; 27, phenylethylamine; 28, α-d-lactose; 29, d,l-α-glycerol phosphate; 30, d-malic acid; 31, putrescine

Carbon source utilization patterns of the external and internal bacterial communities overlapped in most cases within samples from a particular site (Fig. 4). Some discrepancies can be seen in the sample duo (external/internal) L1, where the external community had a higher affinity for metabolizing β-methyl-d-glucoside (106.92 vs 0.78 OAU), γ-hydroxybutyric acid (71.82 vs 0.00 OAU), and l-asparagine (92.48 vs 0.00 OAU), whereas the internal community preferred glycogen (31.55 vs 161.07 OAU). In sample duo L2, the external community exceeded over the internal in the utilization intensity of β-methyl-d-glucoside (153.08 vs 99.48 OAU) and i-erythritol (150.19 vs 0.00 OAU), whereas in L3, glycogen (97.29 vs 47.56 OAU) and Tween 80 (94.81 vs 0.00 OAU) were utilized more efficiently by the external community. In sample L4, α-d-lactose was utilized more efficiently by the external community (95.7 vs 9.67 OAU). In sample LP, several carbon sources were more intensely oxidized by the internal community, most notably α-d-lactose (124.59 vs 218.00 OAU), d-cellobiose (145.11 vs 211.52 OAU), glycogen (139.48 vs 172.37 OAU), l-threonine (79.26 vs 168.22 OAU), α-cyclodextrin (157.74 vs 207.85 OAU), l-phenylalanine (29.70 vs 140.07 OAU), and i-erythritol (174.37 vs 225.22 OAU).

**Fig. 5:**
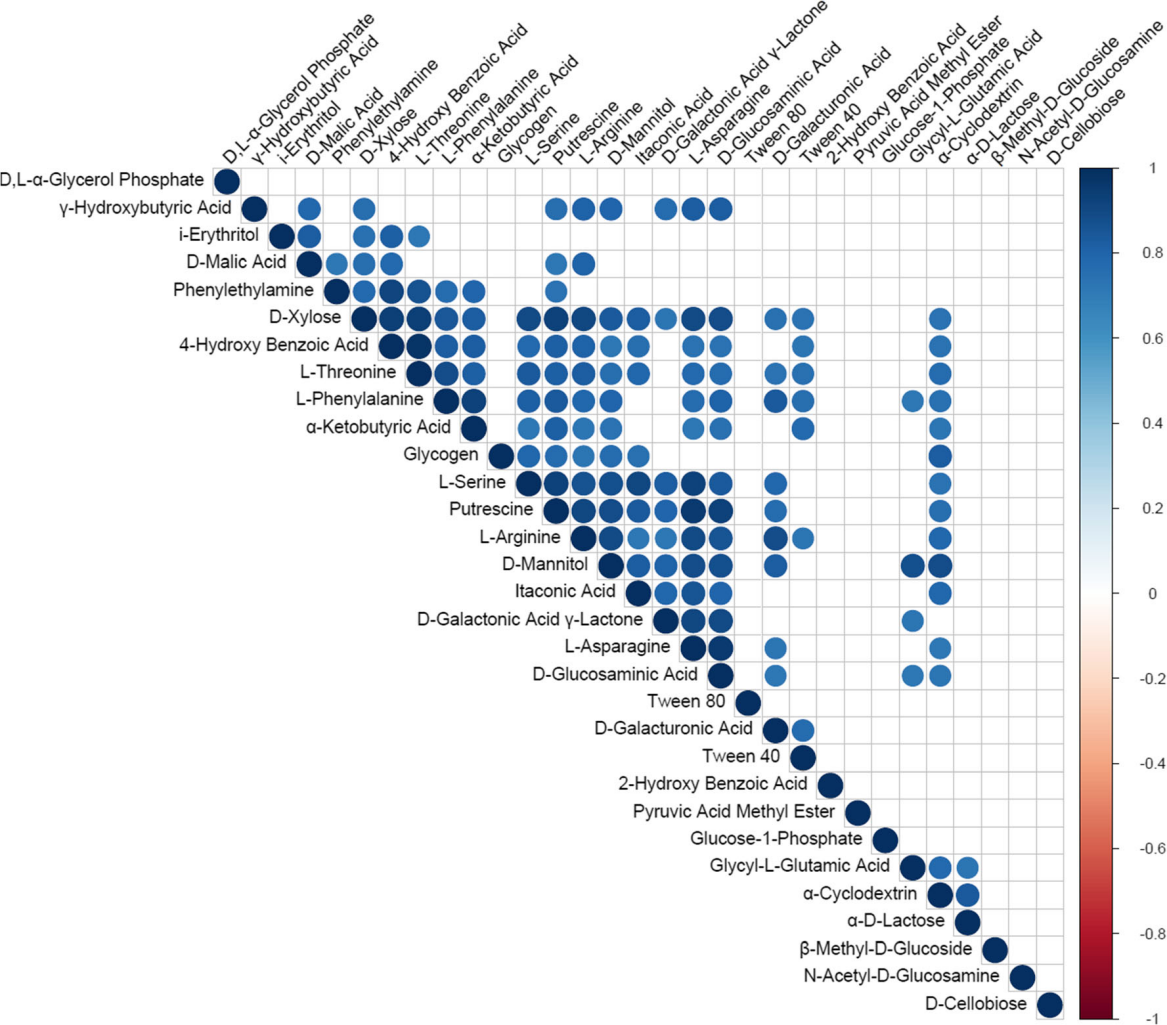
Correlogram of *Leptogium puberulum* bacterial community responses on Biolog EcoPlates. Only significant correlations are shown (*p* ≤ 0.01)

Correlations between carbon source utilization intensity based on the Pearson’s correlation coefficient were all positive (Fig. 5). The strongest correlations observed with the statistical significance of *p* < 0.01 were between the following carbon sources: 4-hydroxy benzoic acid and l-threonine (*r* = 0.98), l-asparagine and d-glucosaminic acid (*r* = 0.96), l-asparagine and putrescine (*r* = 0.95), d-xylose and l-threonine (*r* = 0.94), d-xylose and 4-hydroxy benzoic acid (*r* = 0.93), l-serine and putrescine (*r* = 0.93), l-asparagine and l-serine (*r* = 0.93), and d-xylose and putrescine (*r* = 0.92).

**Fig. 6:**
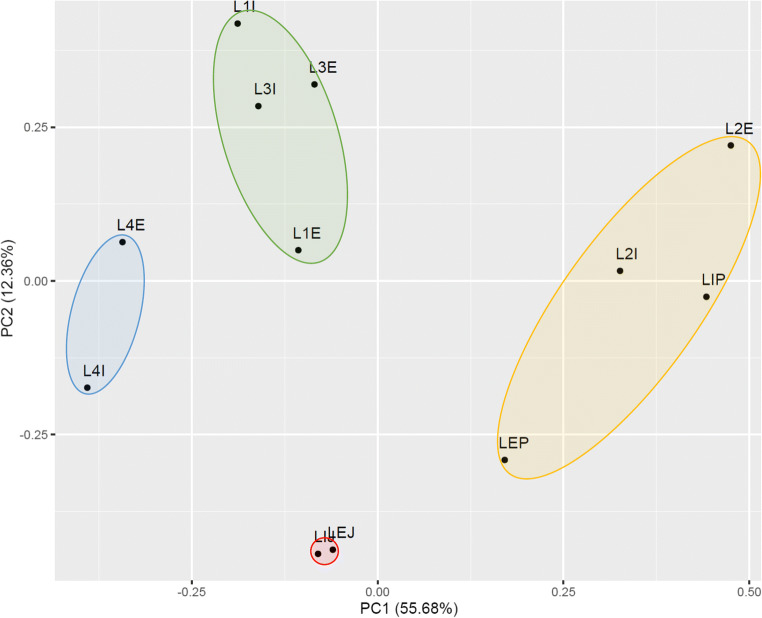
Principal components analysis of *Leptogium puberulum* bacterial community responses on Biolog EcoPlates. E, external community; I, internal community; L1-L4, *Leptogium puberulum* samples of the Ecology Glacier foreland; P, *Leptogium puberulum* samples of Point Thomas penguin rookery; J, *Leptogium puberulum* samples of Jardine Peak area

Principal component analysis (PCA) highlighted four distinct groups within the samples (Fig. 6).The closest clustering was revealed between the communities of samples LEJ and LIJ. Clustering was also apparent between samples L1E, L3E, L3I, and L1I. Samples L4E and L4I formed a separate cluster. Loosely clustered samples LEP, L2I, LIP, and L2E formed the fourth group.

## Discussion

Community-level physiological profiling using Biolog EcoPlates is based on the premise that carbon source diversity of the microbial habitat shapes the catabolic characteristics of the resident bacterial community [19–21]. Substance exchange between the photobiont and the mycobiont is thought to occur largely through specialized hyphae called haustoria, which engulf the cells and bind directly to their cell wall [22]. Data provided in this study, as well as in a paper regarding temperate forest lichens’ bacterial community [23], indicate that a substantial portion of the photosynthesized metabolites may be excreted to the extracellular spaces within the lichen (apoplast) and even outside the thallus boundaries, thus shaping the affected bacterial community. The main photosynthesis product provided to the mycobiont in cyanolichens is d-Glucose [24]. Due to this, the bacterial community associated with the interior and the exterior of the cyanolichen *L. puberulum* catabolized mostly compounds belonging to the carbohydrate group, along with amino acids. Glucose-containing compounds (α-d-lactose, d-cellobiose, glycogen, and α-cyclodextrin) were among the most efficiently metabolized. Furthermore, the sugar alcohol d-Mannitol was also readily catabolized. d-Mannitol was proven to be the main energy-storing compound produced from the acquired photosynthate by lichenized fungi [25]. Seemingly, d-Mannitol was also being released into extracellular lichen spaces, although not necessarily as a nutrient (the photobiont does not metabolize d-Mannitol), [26] but presumably as a compatible solute, a protective measure against desiccation and/or freezing events [25, 27]. Judging by the EcoPlate responses, amino acids may have also been excreted in sufficiently high concentrations by the nitrogen-fixing cyanobiont, to be successfully assimilated and oxidized by the bacterial community. d-Mannitol consumption abilities significantly correlated with amino acid utilization intensity. The *L. puberulum* bacterial community may therefore harbor a portion of species exhibiting a scavenging lifestyle that get enriched in numbers during nutrient surpluses. Some studies also indicate that lichen-associated bacteria can actively degrade lichen thalli components like cellulose and chitin [28]. In this respect, the *L. puberulum* bacterial community expressed d-cellobiose (cellulose derivative) and *N*-acetyl-d-glucosamine (chitin derivative) catabolism, hinting towards their participation in the degradation of the hosts structural components [29]. Utilization of these two sources did not correlate significantly with catabolism of any other compound, pointing towards them as being an innate trait of the lichen associated microbial community, largely independent from the metabolic status of the lichen thalli. The metabolic traits of the chlorolichen *G. regalis* bacterial community were quite similar to the *L. puberulum* community. Despite ribitol being the main export product in *Trebouxia*-containing chlorolichens [30], glucose-bearing compound metabolism was still featured. Some studies indicated that even in those lichens glucose concentrations can be very high, although the underlying mechanism was not explored in detail [31]. However, the most striking feature of the *G. regalis* bacterial community was the very efficient catabolism of fatty acid-containing compounds like Tween40/Tween80. In support of this, lipid droplets and lipid-like substances have been observed in the *G. regalis* ultrastructure collected from the same site [32].

Bacterial metabolic trait changes were also investigated in specimens of *L. puberulum* gathered at the Ecology Glacier forefield across lateral moraines, making up a chronosequence [33], where the distance from the glaciers edge can be substituted for the time since deglaciation. This sampling variant was termed the spatio-temporal gradient. Severe differences in terms of the number of utilized carbon sources, as well as utilization intensity, were apparent in the sampling material. In *L. puberulum* samples procured from the “youngest” site, the bacterial community displayed moderate metabolic diversity on Biolog EcoPlates. This diversity peaked in samples from the neighboring, older site and diminished gradually in samples from sites even further away from the glaciers edge. Considering the dependence of the bacterial community on lichen-derived metabolites [23], this could be explained by the physiological status of the lichen symbiosis, which is strongly connected to lichen development [34]. Several studies on lichen ontogenesis have proposed a scenario of the development of foliose lichens. It was stated that the thallus is mostly active in the marginal, young lobes, while the core remains relatively inert [35]. This is mainly due to the activity of the photobiont, which is dictated by the size, numbers and most importantly, by the age of its cells. Young, small, and numerous cells exhibit high rates of photosynthesis, whereas those of a certain age contribute little to the lichens carbon budget [36]. Therefore, the proportions of the “active” to the “inactive” parts of the thallus can be reflected in the metabolic activity of the bacterial community. Autophototrophs are known to exudate photosynthesis products throughout their cell envelopes when photosynthesis rates are high, in order to: preserve the osmotic and redox potentials of the cell, keep the CO_2_ assimilation going and, in the case of cyanobacteria, the fixation of nitrogen progressing [37]. Consequently, when the amounts and the diversity of the exuded nutrients increases, the bacterial community is enriched in *r* strategists, displaying high rates of respiration and a wide compound assimilation ability [38]. The later decline in metabolic trait numbers can therefore be attributed to the lower output of the ageing photobiont. However, the very low activity of the microbiome in the late stages of *L. puberulum* development is somewhat intriguing. This is the stage, where competition from other plant species is very pronounced [39]. Therefore, the lichen might actively control its resident bacteria to diminish the stripping of essential nutrients and to increase its own competitive value. This could be achieved by means of antimicrobial secondary metabolites [40]. Although cyanolichens usually do not produce such compounds [41], this and other studies merit further research [42]. Indeed, the d-Mannitol and glucose-bearing compound catabolism was largely restricted at this stage, while the consumption of cellulose and chitin digestion derivatives was still active. Some researchers proclaim that bacteria in old lichens degrade the thallus for the benefit of the symbiosis, and that the older parts of this meta-organism get recycled [28].

To assess the influence of nitrogen compound concentration in the lichens’ growth habitat on the catabolic traits of its associated microbiota, specimens of the nitrotolerant *L. puberulum* cyanolichen were collected from sites that profoundly differed in imported labile nitrogen levels, namely, an Adelie penguin nesting site (Point Thomas rookery) and a remote highland plato (Jardine Peak area), where external nutrients are deposited in a limited degree [10]. Lichens growing in the immediate proximity to penguin nesting sites experience labile nitrogen influx via the so-called ammonia shadow—ammonia vapors from the ammonification process of the penguin guano [43]. Metabolic traits of the bacterial community from this area involved catabolism of amino acids and other nitrogen-bearing compounds. A fertilization experiment published in 2003 indicated that high levels of ammonia are converted in N-tolerant lichens into amino acids, mostly l-arginine, to decrease its toxic effects [44]. Moreover, high levels of photobiont derived carbohydrate-like compounds were detected in these fertilized lichens. This could explain the high diversity of metabolic traits, as well as/together with high respiration rates in the samples from the penguin rookery, as high nutrient levels promote highly active *r* strategist proliferation [38], similarly as in highly active samples from the Ecology Glacier foreland, as indicated by the PCA clustering. Additionally, d-malic acid catabolism was very active in the rookery samples. d-malic acid, being a metabolite of the Krebs cycle [45], is also useful in leaching biogenic elements from the substratum [46]. Presumably, nutrients like phosphorus and biometals are among limiting factors in such environmental settings [47]. While sequestering these elements, the lichen also provides an easily palatable carbon source for bacteria [48]. *L. puberulum* growing in the nutrient-restricted area of Jardine Peak harbored a community with a moderate catabolic diversity. An interesting feature in those samples was the suppression of d-cellobiose and *N*-acetyl-d-glucosamine catabolism. This points towards the lack of thalli degradation processes, presumably due to the necessity of maintaining its integrity. In Antarctic settings, having an intact thallus with a large surface area could mean more efficient water and nutrient (dust, sea spray) acquisition, thus possessing better chances in interspecies competition [49]. However, in such oligotrophic conditions, the thallus seems like an easy target for bacterial degradation by resident and soil bacteria [50]. Consequently, preventing thalli destruction may require antimicrobial metabolite secretion [51], which also affects the external microbial community, as highlighted by PCA clustering, presumably to hinder secondary colonization.

## Conclusions

In conclusion, metabolic traits of the *L. puberulum* associated bacteria sampled at the diverse landscape of the western shore of the Admiralty Bay region (King George Island, Antarctica) displayed substantial differences between sampling sites. In general, the *L. puberulum* bacterial community catabolized photobiont- and mycobiont-specific carbon compounds like glucose-containing carbohydrates (α-d-lactose, d-cellobiose, glycogen and α-cyclodextrin) and d-Mannitol. The bacteria also had the ability to process lichen thalli component degradation products (d-cellobiose and *N*-acetyl-d-glucosamine). It was apparent that in situations where lichen metabolism was adjuvated, presumably due to increased photobiont output or external “fertilization,” the bacterial community responded in increased metabolic diversity and respiration intensity. In specimens from older proglacial sites, or in those growing in nutrient-limited conditions, the opposite was the case. Some metabolic traits, like labile carbon-source scavenging or features connected to thalli degradation, were lacking, presumably constricted by the lichen itself (via antimicrobial compound secretion), due to survival/competition related issues. Such phenomena brand the environment-lichen-microbiome interactions as highly complex and worth further attention of multidisciplinary research teams.

## Data Availability

Upon request

## Materials Availability

Upon request

## Code Availability

Upon request
